# Incremental short daily home hemodialysis: a case series

**DOI:** 10.1186/s12882-017-0651-1

**Published:** 2017-07-05

**Authors:** Stephanie M. Toth-Manikowski, Surekha Mullangi, Seungyoung Hwang, Tariq Shafi

**Affiliations:** 1 Division of Nephrology, Johns Hopkins University School of Medicine, 301 Mason Lord Drive, Suite 2500, Baltimore, MD 21224 USA; 2 Welch Center for Prevention, Epidemiology and Clinical Research, Johns Hopkins University, Baltimore, MD USA; 3 Department of Epidemiology, Johns Hopkins Bloomberg School of Public Health, Baltimore, MD USA

**Keywords:** Residual kidney function, Incremental hemodialysis, Home hemodialysis, Uremic toxins, Kt/V, NxStage

## Abstract

**Background:**

Patients starting dialysis often have substantial residual kidney function. Incremental hemodialysis provides a hemodialysis prescription that supplements patients’ residual kidney function while maintaining total (residual + dialysis) urea clearance (standard Kt/Vurea) targets. We describe our experience with incremental hemodialysis in patients using NxStage System One for home hemodialysis.

**Case presentation:**

From 2011 to 2015, we initiated 5 incident hemodialysis patients on an incremental home hemodialysis regimen. The biochemical parameters of all patients remained stable on the incremental hemodialysis regimen and they consistently achieved standard Kt/Vurea targets. Of the two patients with follow-up >6 months, residual kidney function was preserved for ≥2 years. Importantly, the patients were able to transition to home hemodialysis without automatically requiring 5 sessions per week at the outset and gradually increased the number of treatments and/or dialysate volume as the residual kidney function declined.

**Conclusions:**

An incremental home hemodialysis regimen can be safely prescribed and may improve acceptability of home hemodialysis. Reducing hemodialysis frequency by even one treatment per week can reduce the number of fistula or graft cannulations or catheter connections by >100 per year, an important consideration for patient well-being, access longevity, and access-related infections. The incremental hemodialysis approach, supported by national guidelines, can be considered for all home hemodialysis patients with residual kidney function.

## Background

Over a million people are expected to initiate hemodialysis in the US in the next decade [1]. Home dialysis is widely considered to be the preferred dialysis modality, with some estimates considering >25% of the incident patients eligible for home dialysis [2]. However, in 2014 only 7.8% of the 115,363 incident dialysis patients in the U.S. initiated dialysis using a home modality and only 0.3% of all patients were started on dialysis using home hemodialysis [1]. NxStage System One (NxStage Medical, Inc., Lawrence, MA) is the most widely used short daily home hemodialysis system in the US and hemodialysis using this system is typically prescribed 5 days per week.

Most patients starting dialysis have substantial residual kidney function that can contribute to solute clearance and volume homeostasis [3]. Presence of residual kidney function is independently associated with improved survival and quality of life in incident hemodialysis patients [4, 5]. Incremental hemodialysis is an individualized hemodialysis prescription that adjusts the dialysis dose (dialysis Kt/Vurea) by accounting for residual kidney function (residual Kt/Vurea) [6–8]. In patients with significant residual kidney function receiving in-center hemodialysis, incremental hemodialysis prescription is associated with similar survival but longer preservation of residual kidney function, compared to routine thrice weekly hemodialysis [9]. However, there are no data on incremental hemodialysis prescription for home hemodialysis patients in the present era. The flexibility of home hemodialysis scheduling and the substantial residual kidney function in incident hemodialysis patients makes incremental home hemodialysis prescription particularly attractive in this population [3].

**Fig. 1:**
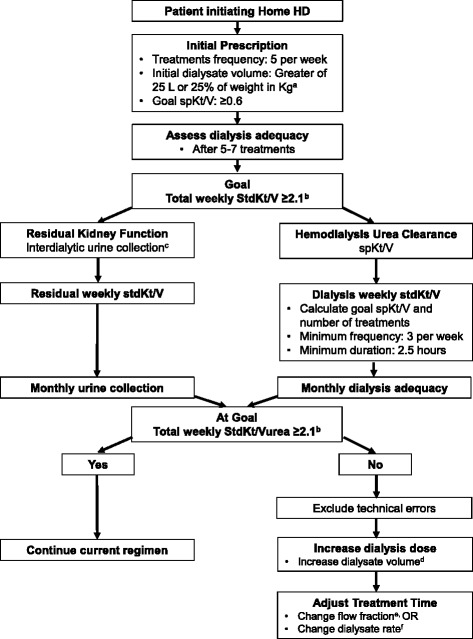
Protocol for prescribing incremental home hemodialysis using NxStage System One. ^a^With the NxStage System S, we now initiate training for all patients using 60 L dialysate per treatment. ^b^Goal stdKt/V is now 2.3 based on KDOQI 2015 Hemodialysis Adequacy Guidelines [39]. ^c^A 12-h collection immediately prior to the monthly urea kinetic modeling session is also an option. ^d^Change in dialysate volume = current dialysate volume x (1 – (current spKt/V/goal spKt/V)). ^e^Flow fraction = effluent rate (dialysate rate plus ultrafiltration rate) / blood flow rate. ^f^Total volume of dialysate + ultrafiltration in L/Desired treatment time [maximum rate is 12 L per hour (200 ml/min) for NxStage System One and 18 L per hour (300 ml/min) for NxStage System S]

Starting 2011, we implemented an incremental home hemodialysis protocol at a home hemodialysis unit affiliated with Johns Hopkins University in Baltimore, Maryland (Fig. 1). In this case series, we describe the characteristics, dialysis dosing, laboratory parameters, and outcomes of the first five patients with residual kidney function treated with incremental home hemodialysis using NxStage System One.

## Case presentation

We retrospectively identified patients treated with incremental hemodialysis and abstracted their data from the electronic medical record. We followed patients from the start of hemodialysis in our center to the end of the study period (December 17, 2015), transfer to another unit, kidney transplantation or death. The Johns Hopkins Medicine Institutional Review Board reviewed and approved this study with waiver of consent for the retrospective review.

The protocol for incremental home hemodialysis is presented in Fig. 1. We measured residual renal function using a 24-h urine collection immediately preceding the dialysis session for monthly labs. The central laboratory for the large dialysis organization uses a factor of 0.9*predialysis-urea level to calculate the residual urea clearance and residual stdKt/Vurea. The laboratory also reports dialysis stdKt/Vurea calculated using data from the modeling session and the number of prescribed dialysis treatments, using equation recommended by the 2006 KDOQI hemodialysis adequacy guidelines [10]. We calculated the total weekly stdKt/Vurea as the sum of residual stdKt/Vurea and dialysis stdKt/Vurea. In general, when a new dialysate volume was calculated, we rounded it up to optimize the use of dialysate bags or the prepared dialysate batch. On NxStage System One, the treatment time is mainly determined by the dialysate volume, blood flow rate, and ultrafiltration volume. Since the blood flow rate is generally constant, the dialysate volume determines the treatment time, which can be modified by adjusting the flow fraction (effluent rate/blood flow rate). Higher flow fraction increases the dialysate flow rate and shortens treatment time. The effluent rate (dialysate rate + ultrafiltration rate) is the limiting factor in this step [maximum of 12 L/h (200 ml/min) for NxStage System One and 18 L/h (300 ml/min) for NxStage System S].

We treated 5 incident dialysis patients with residual kidney function using this incremental regimen. Patient characteristics, dialysis parameters, and laboratory test results are presented in Table 1. Of the two patients with follow-up >6 months, residual kidney function was preserved for ≥2 years. The biochemical parameters of all patients remained stable. Importantly, the patients were able to transition to home hemodialysis without automatically requiring 5 sessions per week at the outset and gradually increased the number of treatments and/or dialysate volume as the residual kidney function declined. A brief description of the individual patients follows:

**Table 1:** Patient and home hemodialysis characteristics

Patients		Dialysis		Urine			Blood				Total stdKt/V	Other labs				
		Treatments per week	Dialysate volume, L (Per treatment)	Volume, mL/day	Urea Clearance, mL/min	Renal stdKt/V	BUN pre, mg/dL	URR, %	spKt/V	Dialysis stdKt/v		K	CO2	Phos	Albumin	Hb
Patient 1	Baseline	5	25	1000	3.02	0.72	49	35	0.47	1.79	2.51	5	19	5.2	4.1	8.4
	6 months	4	25	750	2.67	0.65	80	43	0.63	1.74	2.39	4.2	23	4.9	4.3	9.8
	12 months	4	25	750	2.58	0.65	82	40	0.6	1.69	2.34	4.7	23	4.5	4.0	9.9
	18 months	4	25	500	1.78	0.44	91	43	0.62	1.70	2.14	4.1	21	4.6	4.1	9.6
	24 months	4	30	750	1.56	0.38	91	45	0.77	2.00	2.38	4.5	21	5.1	4.2	10.4
	30 months	4	30	0	0	0	92	49	0.82	2.09	2.09	5.2	23	4.2	4.2	10
Patient 2	Baseline	3	20	1600	4.1	1.19	58	45	0.67	1.33	2.52	4.7	18	4.6	4.4	10.7
	6 months	3	20	1500	5.41	1.57	60	47	0.71	1.38	2.95	5.8	20	6.4	4.2	10
	12 months	4	20	750	2.23	0.57	89	43	0.61	1.65	2.22	5.1	22	5.7	3.8	10.6
	18 months	4	20	800	2.13	0.58	45	42	0.54	1.50	2.08	4.4	28	4.2	4.3	9.2
	24 months	4	30	750	1.16	0.32	49	51	0.78	1.94	2.26	4.6	24	4.5	4.0	9.8
Patient 3	Baseline	3	20	700	5.57	1.05	54	37	0.54	1.12	2.17	3.1	23	3.5	4.0	8.2
	6 months	4	20	500	3.07	0.7	75	33	0.48	1.39	2.09	3.8	27	3.9	3.8	8.2
Patient 4	Baseline	5	30	2200	11.5	1.66	43	40	0.49	1.87	3.53	4	28	4.3	2.6	9.1
	6 months	5	30	1000	3.95	0.56	82	23	0.38	1.53	2.09	6.1	24	6.5	3.5	8.5
Patient 5	Baseline	3	20	750	6.98	1.38	34	35	0.48	1.01	2.39	3.8	27	3.2	3.7	11.4
	6 months	3	25	750	5.47	1.08	32	50	0.74	1.43	2.51	3.2	25	4	3.6	9.6

*Abbreviations:* *BUN* blood urea nitrogen, *URR* urea reduction ratio, *K* potassium, *CO2* bicarbonate, *Hb* hemoglobin, *Phos* phosphate

Patient 1: This patient initiated home hemodialysis training using NxStage System One 5 days per week. The baseline 24-h urine volume was 1000 mL/day with a urinary urea clearance of 3 mL/min corresponding to a weekly renal stdKt/V of 0.72. Hemodialysis frequency was decreased to 4 treatments per week. Over time, as the residual urea clearance declined, dialysis urea clearance was increased by increasing the dialysate volume. By using this incremental approach to dialysis, we were able to achieve urea clearance targets with 4 treatments per week, instead of 5 treatments per week, which would have been necessary if we ignored residual function. As a result, the patient was able to avoid >200 cannulations of the fistula over a period of 2 years (1 less treatment per week equals 2 less cannulations per week * 52 weeks * 2 years). Reducing the number of cannulations was an important quality of life consideration for this patient, which we were able to achieve using an incremental regimen.

Patient 2: This patient was transferred to the home hemodialysis program from recently initiated in-center hemodialysis. The initial urine volume was 1600 mL/day with a urinary urea clearance of 4.1 mL/min and a weekly renal stdKt/V of 1.19. Home hemodialysis was started using NxStage System One with 3 treatments per week schedule (every other day). The patient maintained residual kidney function until undergoing kidney transplantation 2 years after hemodialysis initiation. Using an incremental approach, allowed this patient to avoid >200 cannulations while undergoing home hemodialysis (1 less treatment per week equals 2 less cannulations per week * 52 weeks * 2 years).

Patient 3: This patient underwent timed urine collection prior to hemodialysis initiation. The initial urine volume was 700 mL/day with a renal stdKt/V of 1.05. Home hemodialysis was initiated with 3 treatments per week, increasing to 4 per week as the residual kidney function declined. Follow-up data is only reported to 6 months as contractual issues led to closure of the clinic where the patient was under treatment.

Patient 4: This was a morbidly obese patient who started home hemodialysis for intractable volume overload. The initial urine volume, on diuretics, was 2200 mL/day with a urinary urea clearance of 11.5 mL/min and renal stdKt/V of 1.66. Dialysis was initiated using home hemodialysis with a 5 days per week regimen due to volume overload. Using an incremental approach allowed the patient to maintain adequate clearance with the use of a lower dialysate volume (30 L), with a shorter treatment time than what would have been required if residual kidney function was ignored. At 6 months after dialysis initiation, the patient moved out of the area due to employment reasons and was transferred to another home hemodialysis program.

Patient 5: This patient was on in-center maintenance hemodialysis who sought personalized care as the patient had noticed significant urine volume. The baseline urine volume was 750 mL/day with a urinary urea clearance of 6.98 mL/min and weekly stdKt/V of 1.38. The patient started home hemodialysis with 3 times per week schedule (every other day). The follow-up data is only reported to 6 months as contractual issues led to closure of the clinic where the patient was under treatment.

## Discussion

There are several key points highlighted by this case series. First, an incremental regimen can improve patient acceptability of home hemodialysis and make the transition to home hemodialysis easier. It allows for titration of dialysis dose on an individual basis and enables patients to remain in control over how and when they perform hemodialysis in the home, very different from the one-size-fits-all approach used for patients undergoing thrice-weekly in-center hemodialysis. Second, less frequent cannulation of vascular access means lowering the number of access cannulations by >100 per year (assuming 1 less treatment per week equals 2 less cannulations per week * 52 weeks per year). This is an important consideration for patients’ quality of life by reducing pain and discomfort with cannulation, and it may also reduce the risk of access complications and blood stream infections. Third, the preserved residual kidney function in the two patients with >6 months of follow-up is encouraging. It is interesting to note that the NxStage System uses ultrapure dialysate which has been associated with reduced inflammation and slower residual kidney function decline [11–13]. Applying an incremental approach to home hemodialysis offers a unique advantage to the patient on various fronts. It is tantamount to personalized medicine for the dialysis patient and equips the nephrologist with a patient-centered approach to what has historically been a rather regimented thrice-weekly hemodialysis prescription. Additionally, it has the potential to reduce cost of care to both the patient and the healthcare system without affecting dialysis adequacy.

The importance of residual kidney function preservation cannot be emphasized enough [14–16]. While it is well known that residual kidney function is independently associated with improved survival, patients also report improved healthcare-related quality of life, have better volume and blood pressure control, improved phosphate and potassium clearance, and reduced erythropoietin requirements. Perhaps most importantly, native kidney function allows for excretion of uremic solutes that hemodialysis does not adequately remove [17]. Many of these uremic solutes are potential uremic toxins that contribute to morbidity and mortality in hemodialysis patients [4, 18–20].

Previous observational studies suggest that twice-weekly hemodialysis using an incremental approach to hemodialysis initiation results in preserved residual kidney function [9, 21, 22]. Incremental hemodialysis is widely practiced outside the U.S. [22–34] and is now gaining acceptability in the U.S. [35]. While the benefits of frequent hemodialysis on volume overload and left ventricular hypertrophy are well described in anuric prevalent hemodialysis patients, [36] there remain concerns with the effect of frequent hemodialysis in incident hemodialysis patients with residual kidney function. In the Frequent Hemodialysis Network Trials, more frequent hemodialysis was associated with a faster loss of residual kidney function [37]. Additionally, benefit of frequent hemodialysis on left ventricular mass reduction was most evident in patients without residual kidney function at baseline; 8 of 9 patients with a pronounced reduction in LV mass in the Daily Trial were anuric [38]. Frequent hemodialysis also increased the risk of access complications, [39] prompting caution in the 2015 KDOQI hemodialysis adequacy guidelines [40]. These findings call into question the conventional approach of one-size-fits-all dialysis regimen without consideration of residual kidney function, particularly in incident hemodialysis patients [3, 7].

One practical limitation to the use of incremental home hemodialysis is the requirement of monthly timed urine collections. Although the KDOQI guidelines recommend these measurements to be done quarterly if incremental dialysis is prescribed, [10] we elected to monitor it monthly to ensure adequate solute clearance. All patients were quite willing to perform monthly 24-h urine collections as the results had meaningful impact on their treatment. Generally, the timed urine collection is done over the entire interdialytic interval with blood samples for urea collected at the end of the hemodialysis treatment when urine collection is started and at the beginning of the next hemodialysis treatment when urine collection ends [10]. However, there are several limitations of this technique including its onerous nature and chances for errors during collection. Additionally, residual kidney function is not at steady state in the interdialytic period. It is the lowest at the end of hemodialysis, presumably from volume depletion, and the highest at the end of the interdialytic interval, as shown in an elegant study by van Olden et al. [41]. These authors recommended that the best approach to measuring residual kidney function may be timed urine collection for 12 h prior to start of hemodialysis and a single blood sample at the end of urine collection interval (start of hemodialysis) [41]. An alternative approach without requiring urine collection is to estimate residual kidney function from serum markers, similar to estimating GFR from serum creatinine [42]. The low molecular weight proteins such as β-trace protein (BTP), may be used for estimating residual kidney function in patients undergoing standard high-flux hemodialysis (online calculator: http://www.ureaclearance.org/). BTP is available for clinical use in Europe, but not in the U.S. at the present time.

## Conclusions

Our small case series demonstrates that incremental hemodialysis can be safely used to individualize a dialysis dose and preserve residual kidney function in patients undergoing home hemodialysis. Although our case-series included a small number of patients, the results were gathered in a real-life clinical setting, and are therefore generalizable to any patient with residual kidney function starting home hemodialysis. Our study adds to a growing body of literature on the use of incremental hemodialysis in the contemporary time period in the U.S. and the rest of the world.

In conclusion, incremental home hemodialysis prescription is simple, feasible, and can be safely prescribed for patients with residual kidney function. The flexibility of home hemodialysis scheduling and the potential benefit of incremental hemodialysis on preservation of residual kidney function makes this an attractive treatment option for patients initiating dialysis.

## Abbreviations

BTP: β-trace protein
ESRD: End-stage renal disease
KDOQI: Kidney disease outcomes quality initiative
